# Understanding the role of zinc ions on struvite nucleation and growth in the context of infection urinary stones

**DOI:** 10.1093/mtomcs/mfae017

**Published:** 2024-04-10

**Authors:** Jolanta Prywer, Agnieszka Torzewska, Ewa Mielniczek-Brzóska

**Affiliations:** Institute of Physics, Lodz University of Technology, ul. Wólczańska 217/221, 93-005 Łódź, Poland; Department of Biology of Bacteria, Faculty of Biology and Environmental Protection, University of Lodz, ul. Banacha 12/16, 90-237 Łódź, Poland; Institute of Chemistry, Faculty of Science and Technology, Jan Długosz University of Czestochowa, ul. Armii Krajowej 13/15, 42-200 Częstochowa, Poland

## Abstract

Taking into account that in recent decades there has been an increase in the incidence of urinary stones, especially in highly developed countries, from a wide range of potentially harmful substances commonly available in such countries, we chose zinc for the research presented in this article, which is classified by some sources as a heavy metal. In this article, we present the results of research on the influence of Zn^2+^ ion on the nucleation and growth of struvite crystals—the main component of infection urinary stones. The tests were carried out in an artificial urine environment with and without the presence of *Proteus mirabilis* bacteria. In the latter case, the activity of bacterial urease was simulated chemically, by systematic addition of an aqueous ammonia solution. The obtained results indicate that Zn^2+^ ions compete with Mg^2+^ ions, which leads to the gradual replacement of Mg^2+^ ions in the struvite crystal lattice with Zn^2+^ ions to some extent. This means co-precipitation of Mg-struvite (MgNH_4_PO_4_·6H_2_O) and Zn*_x_*-struvite (Mg_1-_*_x_*Zn*_x_*NH_4_PO_4_·6H_2_O). Speciation analysis of chemical complexes showed that Zn*_x_*-struvite precipitates at slightly lower pH values than Mg-struvite. This means that Zn^2+^ ions shift the nucleation point of crystalline solids towards a lower pH. Additionally, the conducted research shows that Zn^2+^ ions, in the range of tested concentrations, do not have a toxic effect on bacteria; on the contrary, it has a positive effect on cellular metabolism, enabling bacteria to develop better. It means that Zn^2+^ ions in artificial urine, *in vitro*, slightly increase the risk of developing infection urinary stones.

## Graphical Abstract

**Graphical Abstract:**
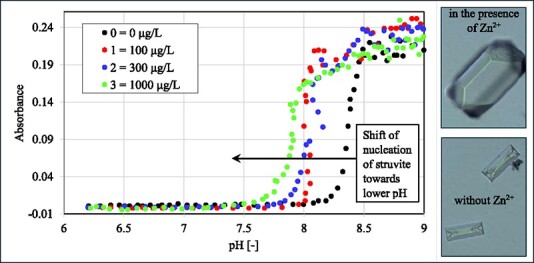
Zinc Zn^2+^ ions in artificial urine in the range of tested concentrations slightly shift the formation of struvite towards lower pH. At the same time, in the presence of Zn^2+^ ions, struvite crystallizing in the lower pH range is larger and has a richer morphology compared to the control test (without Zn^2+^ ions).

## Introduction

**Fig. 1:**
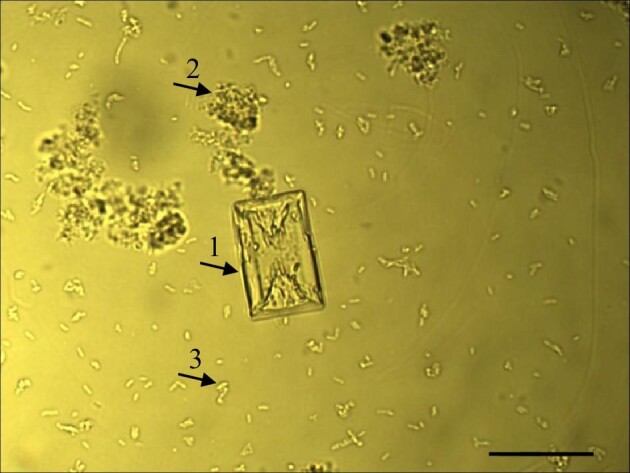
Struvite (arrow 1) and PCaAP (arrow 2), in an artificial urine, induced to growth by *P. mirabilis* (arrow 3). Scale bar: 40 μm.

The main component of infection urinary stones is magnesium ammonium phosphate hexahydrate (MgNH_4_PO_4_·6H_2_O), i.e. struvite, which is highly crystalline (arrow 1 in Fig. 1). In addition to struvite, other more or less crystalline and/or amorphous solid phases are also identified in infection urinary stones. These phases include but are not limited to carbonate apatite, hydroxyapatite, amorphous calcium carbonate, amorphous calcium phosphate, and/or amorphous calcium carbonate.^1–4^ Generally, these phases are called poorly crystalline and amorphous precipitate (PCaAP).^5^ These phases are marked with arrow 2 in Fig. 1. For these components of infection urinary stones (struvite and PCaAP) to form, there must be urease-positive bacteria (arrow 3 in Fig. 1) in the human urinary tract. The most common bacteria are *Proteus, Staphylococcus, Pseudomonas, Providencia*, and *Klebsiella*.^6–8^ Under the influence of urease, bacteria decompose urea—one of the main components of urine—into ammonia and carbamic acid. This initiates a whole series of chemical reactions, which in turn lead to the crystallization of struvite and the formation of PCaAP, which is accompanied by an increase in pH.^5^ For PCaAP, the pH value at which crystallization begins is 6.2, in the case of struvite, it is 7.2.^6^ The exact course of chemical reactions can be found in the literature (e.g. Refs^9–14^). The formation of struvite and PCaAP under the influence of bacterial activity can be classified as a process known as bacteria-induced mineral precipitation. Such precipitation of minerals, depending on the species of bacteria and the environment in which it takes place, is quite common. Minerals that are precipitated due to bacterial activity include, e.g. tricalcium phosphate Ca_3_(PO_4_)_2_, bobierrite Mg_3_(PO_4_)_2_·8H_2_O, baricite (MgFe)_3_(PO_4_)_2_·8H_2_O, and vivianite Fe_3_(PO_4_)·2H_2_O.^15^ All these minerals are classified as phosphates, just like struvite. However, under the influence of bacteria, carbonates, silicates, sulphides, sulphates, oxides, and others can also be precipitated.^15^

In recent years, there has been a trend indicating an increase in the incidence of urinary stones, especially among people living in highly developed countries.^15–18^ This also applies to infection stones. The estimated percentage of people struggling with this disease reaches up to several dozen %,^19^ depending on the geographical region, and has been constantly increasing in recent years (e.g. Refs^20–22^). It is therefore worth asking what factors are associated with the progressive increase in the incidence of infection stones in highly developed countries. The starting point is the assumption that in highly developed countries, human may be exposed to various unfavourable factors originating from the environment and the way of life. Many of these factors may also influence susceptibility to urinary tract infections and, consequently, the development of infection stones. For the research included in this article, from a wide range of such factors, we chose zinc, which is classified as a heavy metal. In this article, we evaluate the effect of Zn^2+^ ion on the nucleation and growth of struvite crystal—the main component of infection urinary stones. Struvite growth is carried out in the environment of artificial urine in two experimental systems. In the first system, the presence of bacteria and the decomposition of urea is simulated by adding an aqueous solution of ammonia (NH_3_·H_2_O), and in the second system, crystallization takes place in the presence of appropriate bacteria.

Zinc is a blue–white, brittle metal. In living organisms, it most often occurs in the form of the Zn^2+^ ion and plays the role of an important trace element. It is believed to have many health-beneficial functions, such as: increasing the immune response to numerous infectious agents, including viruses, and accelerating wound healing. The recommended amount of zinc that should be supplied with food is 11.0 mg/day for men and 9.4 mg/day for women.^6,23^ The best sources of zinc are products of animal origin, such as: oysters (91 mg/100 g), beef (6.8 mg/100 g), pork (3.5 mg/100 g), and Cheddar cheese (3.1 mg/100 g). In the case of products of plant origin, the zinc content per 100 g ranges from 1 to 2 mg.^24^ The Zn^2+^ ion can also be absorbed by living organisms through direct contact with the skin or fumes of this metal.^25^ It is therefore not surprising that people in developed countries, having access to a large number of meat dishes and dietary supplements, are able to provide their bodies with larger amounts of this ion than the populations of developing and poorer countries. In addition, with a vegan diet or one low in animal products, ensuring the appropriate level of the trace element zinc may be a challenge.

Zinc resources in the adult human body are 1.5 g for women and about 2.5 g for men.^26,27^ Zinc found in organisms is usually divided into two pools. The first one is zinc found in the skeletal system, i.e. in muscles and bones, it is estimated that it is about 80%–90% of the total amount of this element in our bodies. These stocks are slowly replaceable, sources say it takes several weeks to replace this pool. The remaining 10%–20% is zinc, which our body exchanges quickly (several days), this pool is used for biological purposes. This zinc (quickly exchangeable) is found mainly in the liver, pancreas, and in the walls of the digestive tract. This resource is sensitive to the amount of zinc supplied with food. Absorption from the digestive system in adults is usually in the range: 15%–50%.^23,27,28^ However, it happens (in the case of severe deficiencies) that the absorption increases to 90%.^23^ At the same time, it is worth noting that zinc in aqueous solutions is more easily absorbed than that found in solid foods.^29^ In the states of zinc deficiency in the human body, its amount in the feces and urine drops sharply, the body is able to absorb part of the zinc that was intended to be excreted from the intestine, if the amount supplied with food is insufficient. The main organs responsible for zinc absorption are small intestine and large intestine. It is from the intestinal lumen that the body absorbs the ion supplied from food, which then goes to the blood, from where it is transported to the organs that need it. The intestines are also responsible for the excretion of unnecessary zinc from the body to the greatest extent. It has been established that fecal excretion accounts for about 90% of the total amount of zinc that the body gets rid of. Other significant channels for getting rid of this element are urine and sweat. It is estimated that the amount of zinc excreted in the feces ranges from 1 to 5 mg per day, and the amount of zinc excreted in the urine ranges from 100–1000 μg/l.^23–25,28,30,31^

In this article, we present research on the effect of Zn^2+^ ions on the nucleation and growth of struvite—the main component of infection urinary stones. The following three Zn^2+^ ion concentrations, along with a control sample (concentration 0–no zinc; 0 μg/l), are the basis for experimental testing: concentration 1 = 100 μg/l, concentration 2 = 300 μg/l, concentration 3 = 1000 μg/l. A concentration 1 corresponds to the amount of zinc below normal in human urine. Such an amount was observed in people exposed to a low supply of this element in the diet.^27–29^ Concentration 2 is considered normal,^24,28,31^ and concentration 3 is an extremely high value that researchers have measured in some women exposed to zinc fumes while working in an electroplating plant.^32^ These concentrations seem to adequately represent the variability in the amount of zinc in human urine.

## Materials and methods

### Study of struvite crystallization in artificial urine without and with bacteria

The experiments presented in the article were carried out in an artificial urine environment, the composition of which is presented in Table 1.

**Table 1.** Composition of artificial urine^33^

Substance	[g/l]
CaCl_2_ · 2H_2_O (Calcium chloride dihydrate)	0.651
MgCl_2_ · 6H_2_O (Magnesium chloride hexahydrate)	0.651
NaCl (Sodium chloride)	4.6
Na_2_SO_4_ (Sodium sulphate)	2.3
Na_3_C_6_H_5_O_7_ (Trisodium citrate)	0.65
Na_2_C_2_O_4_ (Disodium oxalate)	0.023
KH_2_PO_4_ (Potassium dihydrogen phosphate)	2.8
KCl (Potassium chloride)	1.6
NH_4_Cl (Ammonium chloride)	1.0
CH_4_N_2_O (Urea)	25.0
C_4_H_7_N_3_O (Creatinine)	1.1
tryptic soy broth (TSB)	10.0

The content of minerals in artificial urine of such composition corresponds to the average daily concentration in the urine of a healthy person and is widely accepted in this type of research.^1,34^ Since we focus on struvite nucleation and growth, rather than PCaAP, we did not add the first ingredient in Table 1. Adding this ingredient would crystallize PCaAP. The presence of PCaAP would interfere with the possibility of objective assessment of the progress of struvite crystallization. A detailed description of the formation of struvite and PCaAP in infected urine is presented, e.g. in Refs.^1,35–37^ Artificial urine was prepared by dissolving chemical reagents (Sigma–Aldrich) in deionized water. The solution was then filtered through a membrane filter with a pore size of 0.2 µm and the initial pH was set at 5.8. The artificial urine was stored for a maximum of 48 h at 4°C.

The experiment was carried out in two ways: without the presence of bacteria and in their presence. In the first case, the presence of bacteria was simulated by adding an aqueous ammonia solution, NH_3_·H_2_O, (1.2 M). This addition of an aqueous solution of ammonia raises the pH and provides ammonium ions, which mimics the decomposition of urea into the urine by urease-positive bacteria. This procedure has also been used before—e.g. Refs.^38–40^ The second type of experiment was performed in the presence of *Proteus mirabilis* (*P. mirabilis*) bacteria, which are urease positive, and which have been isolated from human urinary stone. Bacterial strain C11-K was obtained by courtesy of the Second Department of Urology, Medical University of Lodz, Poland and isolated from kidney stone of patient of this clinic. Prior to the experiment, bacteria were cultured on tryptic soy broth abbreviated as TSB (urine component used to stimulate bacterial growth; Table 1) for 24 h at 37°C. A suspension of *P. mirabilis* at a concentration of 5 × 10^5^ CFU/ml (CFU/ml stands for colony forming unit per millilitre) was placed in urine and incubated at 37°C. The experiments, both in the presence of bacteria and without them, were carried out up to pH 9–9.5. This is the highest pH that can be reached in the presence of bacteria. Typically, this pH value is obtained after 24 h of the experiment and does not increase further.^41,42^ The pH level is correlated with the viability of bacteria, pH above 8 has a bactericidal effect.

The pH of the artificial urine was monitored during the experiments using a digital pH meter (Elmetron CPC-401), which is accurate to 0.01. In the article, we present the results averaged with an accuracy of 0.1. The experiments were carried out in thermostatic conditions at the temperature of 37 ± 0.5 °C. The temperature was kept constant by circulating the water in a constant temperature water bath. Experiments were performed three times to assess reproducibility.

In the case of the experiment without bacteria, the amount of crystallized struvite was determined by weighing samples that were dried after the urine had been centrifuged at 4400 rpm for 10 min (5702 Eppendorf). The samples were weighed five times, then the results were averaged, and the standard deviation was calculated. In the case of an experiment in the presence of bacteria, it is not possible to separate the crystallized precipitate from the bacterial suspension. In such a situation, the amount of struvite crystallized is usually determined by the amount of magnesium in the overall sediment containing both bacterial suspension and crystalline sediment. Since magnesium is the main component of struvite, its amount indicates the amount of crystallized struvite. In the case of the experiment presented in this article, the amount of struvite crystallized was determined by the amount of magnesium and zinc in the final sediment containing both bacterial suspension and crystalline sediment (a detailed explanation is provided in paragraph 3.2.1). For these analyses, a sample (1 ml) of crystals with bacterial suspension was centrifuged at 8000 rpm for 10 min (Sigma 1-15). The obtained pellet was suspended in an aqueous solution of nitric acid (30% HNO_3_) and incubated for 60 min at 100°C. After mineralization, concentrations of these ions were determined by atomic absorption spectroscopy (Agilent 240 FS Atomic Absorption Spectrometer). Determinations were performed in an air–acetylene oxidizing flame (13.5 l/min of air and 2 l/min of acetylene) at wavelengths and gap widths of 285.2/0.5 nm and 213.9/1 nm for Mg and Zn, respectively.

To add the Zn^2+^ ion to the samples both without and in the presence of bacteria, a standard solution of zinc chloride—Titrisol 1000 mg Zn^2+^ (ZnCl_2_ in 0.006% HCl solution) was used. Using this standard ZnCl_2_ solution, a solution with a lower concentration was prepared by dilution with water and such a solution was added in the appropriate amount to each of the samples to obtain the Zn^2+^ concentrations selected for testing. Adding a ZnCl_2_ solution to the urine means that chloride ions were also added along with the Zn^2+^ ions. However, chlorine anion is present in the artificial urine in four of its constituents (Table 1), in addition, in quantities expressed in grams per litre, which is orders of magnitude more than the chloride ions added with the Zn^2+^ ions. This means that the amount of chlorine introduced into the solution together with Zn^2+^ ions does not mean significant changes in the concentration of chlorine.

### Spectrophotometric and XRD measurements

In order to evaluate the effect of Zn^2+^ ion of various concentrations on struvite nucleation and growth, the turbidity of artificial urine in the presence of these ions was measured in comparison to the control test (without Zn^2+^ ion). This turbidity was measured as the absorbance of light of a specific wavelength. The optimized wavelength was estimated to be 420 and 600 nm for the experiments without and with bacteria, respectively. The absorbance was measured using a Schimadzu 2600 spectrophotometer (in the absence of bacteria) and an Ultrospec 2000 (Pharmacia Biotech) spectrophotometer in the presence of bacteria using 10 mm optical path length cuvettes. In the absence of bacteria, samples from initial to maximum pH 9.5 in 0.5 increments were taken during all experiments and observed under an Opta Tech MN 800 optical microscope. In the presence of bacteria, samples were taken at regular intervals and observed under a Nikon Eclipse TE2000-S optical microscope. This procedure was designed to evaluate the growth progress of the struvite crystals, their habit, and size.

To assess whether the Zn^2+^ ions introduced into the urine solution incorporated into the structure of struvite crystals, the samples were subjected to X-ray diffraction (XRD) testing. Two samples were tested, the first without the presence of zinc (baseline) and the second sample with the highest concentration of Zn^2+^ ions (1000 μg/l). Both samples free of bacteria. The precipitates appearing during the experiments were taken at pH equal 9. In order to separate the precipitates formed from artificial urine, the samples were centrifuged (a centrifuge 5702 Eppendorf, 4400 rpm for 5 min). Then, the resultant precipitates were rinsed in distilled water three times and each time centrifuged. The prepared precipitates were dried at room temperature for 24 h and examined directly after drying. The prepared dry samples were examined by XRD using an X'pert PRO MPD diffractometer (PANalytical). A Cu K_α_ radiation monochromatized by nickel filter was applied. Measurements were done in the range of 2θ angles from 10° to 90°. A continuous scan was used (step 0.0167°); the measurement time of one step was 30 s.

### The influence of Zn^2+^ ion on the formation of chemical complexes in artificial urine—theoretical analysis

To explain the influence of the Zn^2+^ ion on the nucleation and growth of struvite crystals in artificial urine, a theoretical analysis of chemical complexes formed in urine in the presence of this ion was carried out and the results were compared with the analysis of chemical complexes formed in urine without this ion. This analysis was performed using HySS (Hyperquad Simulation and Speciation) software.^43^ Using the equilibrium constants of complex formation (stability constants) and the equilibrium constants of sparingly soluble salts (solubility product constants) and entering the initial molar concentrations of the complexing ions in the artificial urine, the program allows us to calculate the molar concentrations of different chemical species in a given pH range. Thanks to this software, we can analyse the influence of the Zn^2+^ ion on the formation of chemical equilibria and on the formation of struvite in artificial urine. The stability constants used in the HySS program were calculated using the computer program EQUIL.^44^ Calculations were made for a temperature of 37°C.

### Determination of the influence of Zn^2+^ ions on bacterial viability and urease activity

Simultaneously with pH and absorbance measurements, the number of bacteria and the amount of released ammonia, the end product of urea decomposition by urease, were also determined in the samples.

Ammonia concentration was measured by the phenol hypochlorite colorimetric method.^45^ Briefly, 10 µl of bacterial suspension in synthetic urine was mixed with 100 µl of phenol sodium nitroprusside solution (1% w/v phenol, 0.05% w/v sodium nitroprusside) and 100 µl of sodium hypochlorite solution (0.5% w/v sodium hydroxide, 0.5% w/v sodium hypochlorite) and incubated for 30 min at 37°C. The absorbance value that was measured at 625 nm with a Multiskan Ex (Labsystems, Helsinki, Finland), using all reagents without bacteria as a negative control (blank), was compared with that given by standard solutions of ammonium sulphate.

To determine the number of bacteria in all samples, the bacterial suspensions were serially diluted in saline solution (0.9% NaCl) and aliquots of 100 µl were spread on TSB agar. After overnight incubation at 37°C, colonies on the plates were counted to determine the number of CFU/ml.

## Results

### Effect of Zn^2+^ ion on nucleation and growth of struvite in the absence of urease-positive bacteria

#### Spectrophotometric and XRD analysis

To characterize the processes of struvite nucleation and growth, changes in the turbidity of artificial urine were measured in the absence of Zn^2+^ ions (baseline, symbol 0) and in the presence of Zn^2+^ ions at concentrations of 1 = 100 μg/l, 2 = 300 μg/l, and 3 = 1000 μg/l. First, we present the results obtained without the presence of bacteria. In this case, to mimic the activity of bacteria, and more specifically the activity of bacterial urease, small portions (6 µl) of 1.2 M aqueous ammonia were gradually added to the artificial urine.

**Fig. 2:**
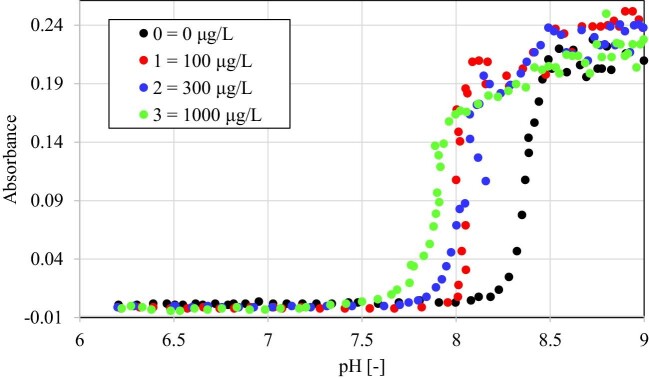
Dependence of absorbance on the pH of artificial urine for the experiment without the presence of bacteria for different concentrations of Zn^2+^ ions given in the inset.

Consequently, as described in the second paragraph of the ‘Materials and methods’ section and many times in the literature,^10–14^ the pH of artificial urine increases and ammonium ions appear and the process of struvite nucleation begins, which is manifested by increasing turbidity of the urine solution. The measure of turbidity is absorbance. The absorbance measurement was performed for the wavelength *λ* = 420 nm. This wavelength was chosen based on the absorption spectrum of struvite in artificial urine compared with the absorption spectrum of a urine sample with the highest concentration of Zn^2+^ ions (1000 μg/l). The absorbance value for this wavelength is the highest for struvite, while the absorbance values measured for the urine solution with zinc are practically zero; therefore, they could not affect the measurement of the absorbance of the three samples with Zn^2+^. The dependence of the absorbance of artificial urine on pH for the baseline (without Zn^2+^ ion) and the tested concentrations of Zn^2+^ ion is shown in Fig. 2.

As can be seen from Fig. 2, in each of the three tested concentrations of zinc (1, 2, and 3), the absorbance started to increase earlier (for lower pH values) than in the case of the control sample (baseline, symbol 0) without zinc, which means that crystallization started earlier in the presence of Zn^2+^ ions. This is indicated by the fact that in the presence of Zn^2+^ ions at lower pH values, compared with the baseline sample, there is a sudden increase in absorbance corresponding to the appearance of struvite. The higher the concentration of zinc, the faster solid phase appears, i.e. for a lower pH. Therefore, the first conclusion is that the Zn^2+^ ion shifts the struvite nucleation point towards lower pH. The maximum absorbance value reached for the highest pH may indicate the number of struvite crystals formed. In the case of the presented studies, the highest value of the maximum absorbance for the highest pH = 9 falls on a sample with a Zn^2+^ concentration of 100 μg/l. For concentration Zn^2+^ of 300 μg/l, the maximum absorbance is slightly lower, and for concentration 3 of 1000 μg/l, the maximum absorbance is even lower, but comparable to the absorbance for the control test (without Zn^2+^ ion). The values of the maximum absorbance for different Zn^2+^ concentrations and the control sample may suggest that the largest number of formed struvite crystals is for the first Zn^2+^ concentration, slightly less for the second concentration, and the least for the third Zn^2+^ concentration. At the same time, the values of the maximum absorbance indicate that for the third concentration of Zn^2+^ and for the control sample, the mass of struvite crystals formed should be comparable. To check whether this is indeed the case, we weighed the resulting crystals. In particular, it was as follows: The artificial urine solution for the highest pH (after the addition of the aqueous ammonia solution) for all tested concentrations 0, 1, 2, and 3 was centrifuged (4400 rpm for 10 min), and then the artificial urine solution was pipetted, leaving centrifuged crystalline sediment at the bottom of the test tubes. After drying, the prepared test tubes were weighed. The crystal precipitate was then removed from the tubes, the tubes were washed and thoroughly dried, and the empty tubes were weighed. Each measurement was performed five times, and the difference in average values between the weight of the tube with and without crystals was taken as the weight of the crystalline precipitate. The masses of the crystalline precipitate obtained are presented in Table 2.

**Table 2.** Final (for pH 9.5) mass of crystalline precipitate [g] for all tested concentrations of Zn^2+^, for an exemplary one-measurement series

Zn^2+^ concentration	Crystalline precipitate mass [g] for pH = 9.5	The percentage [%] increase in weight relative to the baseline
0: baseline: 0 μg/l	0.335 ± 0.001	100
1: 100 μg/l	0.346 ± 0.001	103
2: 300 μg/l	0.353 ± 0.001	105
3: 1000 μg/l	0.314 ± 0.001	94

From Table 2, it is indeed seen that for Zn^2+^ concentrations 1 and 2, the weight of the struvite crystals is slightly higher compared with the control test. On the other hand, for the third concentration of Zn^2+^, the mass of struvite crystals is slightly lower compared with the control test. However, the differences in struvite crystals masses are not large, and it can be assumed that the number of struvite crystals produced is comparable for all tested Zn^2+^ concentrations. Sometimes, the maximum absorbance read from the absorbance vs. pH graph can give misleading information about the amount of crystalline precipitate formed. This happens when the amount of added aqueous ammonia solution is significantly different for individual samples. Then, the same amount of crystalline sediment can be found in samples of different volumes, which gives different values of maximum absorbance. However, it does not seem that such a process occurs in the case of the presented measurements. The amount of aqueous ammonia added to each sample is summarized in Table 3.

**Table 3.** The amount [μl] of added aqueous ammonia solution (1.2 M) for samples with an initial volume of 25 ml for one selected measuring series

Zn^2+^ concentration	Added aqueous ammonia solution [μl]
0: baseline: 0 μg/l	168
1: 100 μg/l	180
2: 300 μg/l	174
3: 1000 μg/l	150

Table 3 shows that the amount of added aqueous ammonia solution is not large and comparable for all samples, which means that based on the maximum absorbance in Fig. 2, it is possible to reliably conclude about the mass of crystalline precipitate formed for individual samples.

**Fig. 3:**
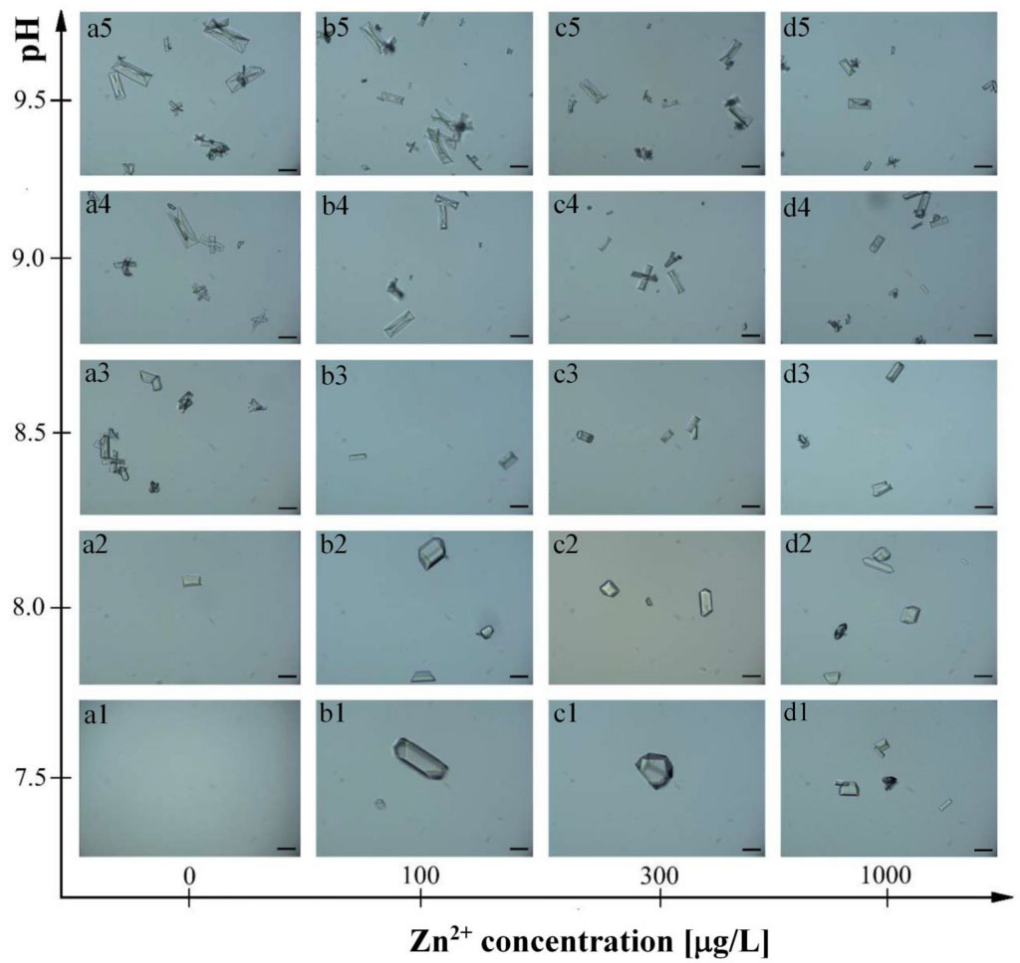
The growth course of struvite crystals in artificial urine at various Zn^2+^ concentrations and increasing pH—a case without the presence of bacteria. Scale bar: 50 µm.

Spectrophotometric measurements (Fig. 2), which show that struvite is formed for lower and lower pH with increasing Zn^2+^ concentration, are confirmed by microscopic observations (Fig. 3). In the case of the baseline, we see the first single crystal at pH = 8, but there is only one crystal in the field of view (Fig. 3, panel a1). For higher pH, the number of struvite crystals increases (Fig. 3, panels a2–a5). The crystals have a coffin-like habit, which is consistent with the literature data.^46–51^ There are also X-shaped penetration twins that are much smaller than coffin-like single struvite crystals. As the pH increases, the number of crystals increases. For Zn^2+^ concentration 1, the struvite growth course is like that of the baseline (Fig. 3, panels b1–b5), except that struvite crystals appear at lower pH. The first crystals are observed already at pH 7.5, and additionally, these crystals are much larger compared with the control test but retain a coffin-like habit. For higher pH, such large struvite crystals no longer exist, but their number systematically increases. For Zn^2+^ concentration 2, the growth of struvite is analogous to that for concentration 1. For low pH, relatively large crystals also occur, which mostly retain a coffin-like habit, but other habits are also found. For higher pH, there are no such large crystals, and the course of struvite growth is analogous to that for the concentration 1 of Zn^2+^, including the appearance of penetration twins. For the concentration 3 of Zn^2+^, for low pH values, such large crystals were not observed as in the case of the concentration 1 and 2 of Zn^2+^. The struvite crystals occurring at this concentration assume a typical coffin-like habit. Penetration twins were also observed for this Zn^2+^ concentration.

**Fig. 4:**
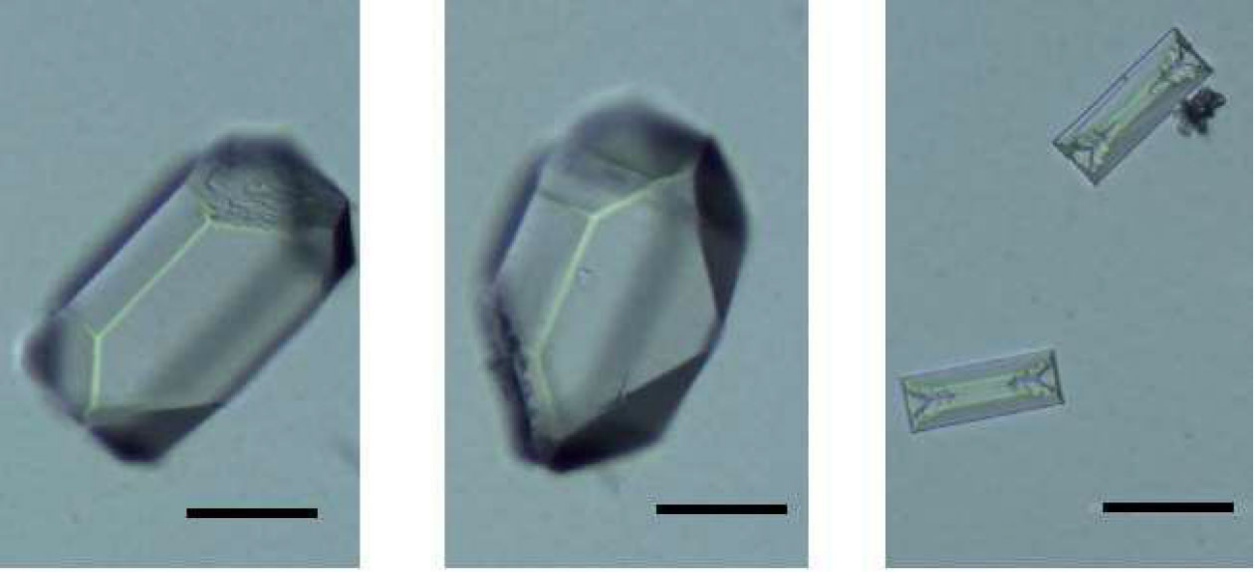
Struvite crystals at pH = 7.5 at Zn^2+^ concentrations of 100 μg/l (left); 300 μg/l (centre) compared with the control test 0 μg/l (right) for pH = 8.5. Scale: 50 μm.

Better visualization of the differences in the size of struvite crystals observed for low pH for Zn^2+^ concentrations 1 and 2 compared with the control test is shown in Fig. 4. Based on Fig. 4, it can be concluded that in addition to the fact that the struvite crystals for low pH and concentrations of zinc 1 and 2 are larger than those in the control test, they also have more faces, but retain an approximately coffin habit. The faces that appear at low pH for zinc concentrations 1 and 2 are corner shear faces.

**Fig. 5:**
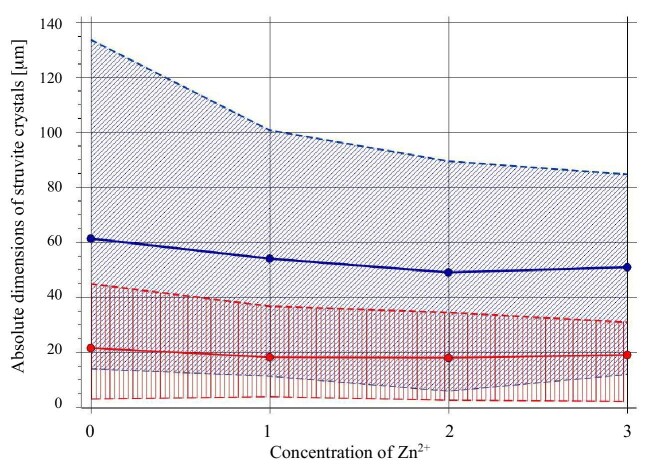
Dependence of the absolute dimensions of struvite crystals along the *a*-axis (red points) and along the *b*-axis (blue points) on the concentration of Zn^2+^: 0 = 0 μg/l, 1 = 100 μg/l, 2 = 300 μg/l, and 3 = 1000 μg/l. The solid lines represent the mean values of the dimensions measured, while the lined areas correspond to span between the measured smallest and largest measured value for a given axis.

A detailed analysis of the absolute dimensions of the crystals is shown in Fig. 5. Specifically, Fig. 5 shows the dependence of the absolute dimensions of struvite crystals along the *a*-axis (red points) and along the *b*-axis (blue points) on the concentration of Zn^2+^ in artificial urine. The solid lines represent the mean values of the dimensions measured, while the lined areas correspond to span between the measured smallest and largest measured value for a given axis. These results were obtained by measuring approximately 50 struvite crystals for each Zn^2+^ concentration and control test. Only those crystals whose location allowed for reliable measurement of both sides (along the *a* and *b* axes) were selected for the measurements. Crystals that formed into clusters or that did not lie perpendicular to the optical axis of the microscope were not measured for fear of inaccurate measurement. Each of the crystals was measured three times (for each axis), and the final result of the side dimension is the average of these measurements presented in Fig. 5. Based on Fig. 5, it can be concluded that for the control sample (Zn^2+^ concentration 0) the crystals have the largest average absolute dimensions compared to the zinc-containing samples. With the increasing concentration of Zn^2+^ ions in the tested samples (concentration of Zn^2+^ 1 and 2), a clear decrease in the absolute dimensions of the crystals can be observed. Only for the extreme concentration of 1000 μg/l, there is a slight change in the trend. A slight increase in the minimum value measured along the *b* axis and in both values of the mean side lengths (along both axes) can be observed.

**Fig. 6:**
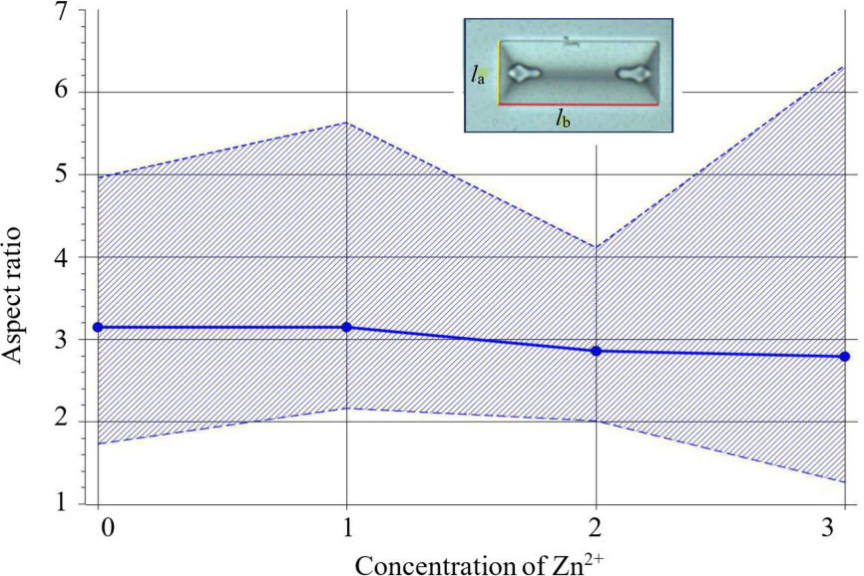
Aspect ratio (*AR*) for different concentrations of the Zn^2+^: 0 = 0 μg/l, 1 = 100 μg/l, 2 = 300 μg/l, and 3 = 1000 μg/l. The solid line corresponds to the average values, and the lined area corresponds to the span between the lowest and highest value of *AR*. The inset defines the lengths *l*_a_ and *l*_b_ of the crystal along the *a* and *b* axes, respectively.

In addition to the absolute dimensions, with increasing Zn^2+^ concentration, the aspect ratio, *AR*, defined as the crystal length *l_b_* along the *b*-axis to the length *l_a_* along the *a*-axis, also changes. The lengths *l_b_* and *l_a_* are defined in inset of Fig. 6: the longer side of the crystal is along the *b*-axis and the shorter side along the *a*-axis. Figure 6 shows the change in *AR* of struvite crystals depending on Zn^2+^ concentration in artificial urine. From Fig. 6, it can be seen that the average *AR* value does not change significantly for concentrations 0 and 1. However, as the Zn^2+^ concentration increases (concentrations 2 and 3), a slight decrease in the average *AR* value can be observed. In the case of the range between the maximum and minimum values for individual Zn^2+^ concentrations, the differences are significant. The largest range is at concentration 3 (the highest concentration of Zn^2+^ ions) and the smallest at concentration 2 (the concentration considered normal in human urine).

In conclusion, the results of the study indicate that in the presence of Zn^2+^ ions in the urine, in which the presence of urease-positive bacteria is simulated by the addition of an aqueous ammonia solution, struvite nucleation and growth occurs earlier (at a lower pH) compared with the control sample (without the presence of Zn^2+^ ions). The amount of struvite formed in the presence of Zn^2+^ ions is slightly higher for concentrations 1 and 2 and slightly lower for the highest concentration 3 compared with the control test (symbol 0). For concentrations 1 and 2 for low pH, struvite is present in the form of crystals much larger than in the control sample. However, as the pH increases, crystals with dimensions comparable to those of the control test are observed. Zinc ions also affect the relative growth rates of individual faces of struvite crystals, as evidenced by the change in the *AR* ratio for the increasing concentration of Zn^2+^ ion.

**Fig. 7:**
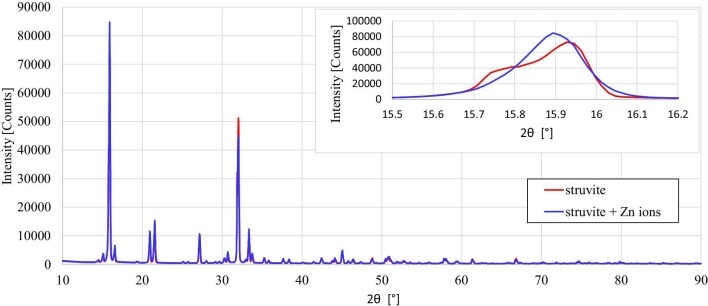
XRD spectra for struvite growing in pure (without the addition of Zn^2+^) artificial urine and struvite growing in artificial urine in the presence of Zn^2+^ ions with the highest concentration 3: 1000 μg/l. The experimental spectrum for struvite (red) is consistent with the reference spectrum for struvite in the International Center for Diffraction Data (ICDD PDF-2 version 2009) card no. 00-015-0762.

One more point is worth considering. It is known from the literature that the growth of a given crystal in the presence of foreign atoms (admixtures) may be disturbed, i.e. the admixture atoms may take nodal or internodal positions instead of proper atoms. Regarding struvite, K-struvite is known, where the K^+^ ion is substituted for the NH_4_^+^ ion.^52^ Such ion exchange is possible because the ionic radii of these ions do not differ much. The question arises whether, in the case of the described experiment, the Zn^2+^ ion, which is a divalent cation, can occupy a nodal position instead of the Mg^2+^ ion, which is also a divalent cation. To check this, we performed XRD tests on two samples: struvite growing in artificial urine for Zn^2+^ concentrations of 0 = 0 μg/l (baseline) and 3 = 1000 μg/l. The results of this measurement shown in Fig. 7 indicate that the obtained diffraction peaks differ in intensity. In addition, the diffraction peaks for struvite growing in artificial urine in the presence of Zn^2+^ ions are shifted relative to the baseline (struvite growing in the absence of Zn^2+^) towards lower 2θ angles. To better visualize this shift towards lower values of 2θ, an enlargement of part of the graph is shown in the inset in Fig. 7. This result means that in addition to struvite with the formula MgNH_4_PO_4_ ·6H_2_O, let us call it Mg-struvite, the tested system can produce Mg_1-_*_x_*Zn*_x_*NH_4_PO_4_·6H_2_O, i.e. struvite with Zn^2+^ ions partially exchanged with Mg^2+^ ions. The value *x* = 1 means that we are dealing with Mg-struvite; *x* = 0 means that all Mg^2+^ ions have been replaced by Zn^2+^ ions and we are dealing with Zn-struvite (ZnNH_4_PO_4_·6H_2_O). If it means that the deficiency of Mg^2+^ ions is compensated by Zn^2+^ ions, which are incorporated into the structure of struvite, and we are dealing with Mg_1-_*_x_*Zn*_x_*NH_4_PO_4_·6H_2_O. Let us call struvite with such partially exchanged ions Zn*_x_*-struvite. The shift of the peaks towards lower 2θ angles generally means that the volume of the Zn-containing unit cell should be larger. Table 4 presents the lattice constants and unit cell volumes of the two tested samples, Mg-struvite and Mg_1-_*_x_*Zn*_x_*NH_4_PO_4_·6H_2_O, calculated on the basis of the obtained XRD spectra. The comparison of these parameters shows that the lattice constants and cell volume of Zn*_x_*-struvite are slightly larger compared with Mg-struvite. This result correlates well with the size of the ionic radii for Mg^2+^ and Zn^2+^, which are 0.72 and 0.74 Å, respectively.^53^ A slightly larger ionic radius of the Zn^2+^ ion consequently results in a slightly larger unit cell.

**Table 4.** Comparison of Mg-struvite and Mg_1-_*_x_*Zn*_x_*NH_4_PO_4_·6H_2_O unit cell parameters (calculated based on *X*-ray powder data, Fig. 7).

	MgNH_4_PO_4_·6H_2_O sample for Zn^2+^ concentration 0 = 0 μg/l (powder)	Mg_1-_*_x_*Zn*_x_*NH_4_PO_4_·6H_2_O sample for Zn^2+^ concentration 3 = 1000 μg/l (powder)
Space group	*Pmn*2_1_	*Pmn*2_1_
*a* (Å)	6.931	6.933
*b* (Å)	6.132	6.131
*c* (Å)	11.195	11.198
*V* (Å^3^)	475.77	476.00
*Z*	2	2

#### Analysis of the results from the point of view of the formation of chemical complexes in artificial urine

To explain the obtained experimental results, both those presented in Fig. 2 and those related to the possible exchange of Mg^2+^ ions for Zn^2+^ ions (Fig. 7), we analysed the chemical complexes formed in artificial urine in the presence of Zn^2+^ ions of different concentrations depending on increasing urine pH compared with the control test (without Zn^2+^ ions). In the artificial urine, both in the case of the control test (without Zn^2+^ ions) and in the presence of Zn^2+^ ions, numerous chemical reactions occur, i.e. dissociation, hydrolysis, and complexation. As a result, the chemical complexes formed create different chemical equilibria. The analysis of these chemical complexes was carried out using the HySS^43^ computer program. The purpose of the analysis is to determine the dominant chemical form of the complexes for baseline—urine with normal composition (Zn^2+^ concentration 0 = 0 μg/l) and in the presence of Zn^2+^ ions of the tested concentrations (concentrations 1, 2, 3). Knowledge of the structure and stability of chemical complexes in a growth solution containing Zn^2+^ ions, is essential to understand the growth kinetics of struvite crystals in the presence of these ions and their influence on the crystal growth processes. Using the analysis of the chemical complexes formed, we would like to obtain information on the mechanism of formation of solid phases in artificial urine for increasingly lower pH with increasing Zn^2+^ ion concentration (Fig. 2). The second interesting thing is the answer to the question whether appropriate chemical complexes are formed, which in turn lead to the formation of Zn*_x_*-struvite.

Table 5 shows the initial concentrations of individual ions, resulting from the composition of the artificial urine and the tested concentrations of Zn^2+^ ions. In the absence of bacteria, their presence is simulated by gradually adding of aqueous ammonia solution as described in point 3.1.1. Table 3 gives the amount of 1.2 M aqueous ammonia solution added. As Table 3 shows, the amount of aqueous ammonia solution added to each measurement series (baseline and in the presence of Zn^2+^ ions) is comparable and does not significantly change the initial sample volume (25 ml).

**Table 5.** Initial concentrations, *c*, of particular ions considered in the HySS calculations

Species	Ca^2+^	Mg^2+^	C_2_O_4_^2−^	Cit^3−^ ^d^	SO_4_^2−^	Na^+^	K^+^	NH_4_^+^	PO_4_^3−^	Zn^2+^
*c* [mM]	0^a^	3.2	0.2	2.5	16.2	126.3	42.0	800^b^	20.67	0^c^
										100 μg/l = 1.53× 10^−3^ mM^c^
										300 μg/l = 4.58×10^−3^ mM^c^
										1000 μg/l = 15.3× 10^−3^ mM^c^

a In this study, we used artificial urine with modified composition without calcium chloride dihydrate, which results in an initial Ca^2+^ concentration of 0 mM.

b This amount of NH_4_^+^ results from the assumption that the entire available amount of urea (25 g/l; Table 1) is decomposed into form, inter alia, NH_3_ in gaseous form (details in Ref.^53^)

c These are the Zn^2+^ concentrations tested in this study—see Table 2.

d Cit stands for [C_6_H_5_O_7_] and results from the presence of trisodium citrate in the artificial urine (Table 1).

**Fig. 8:**
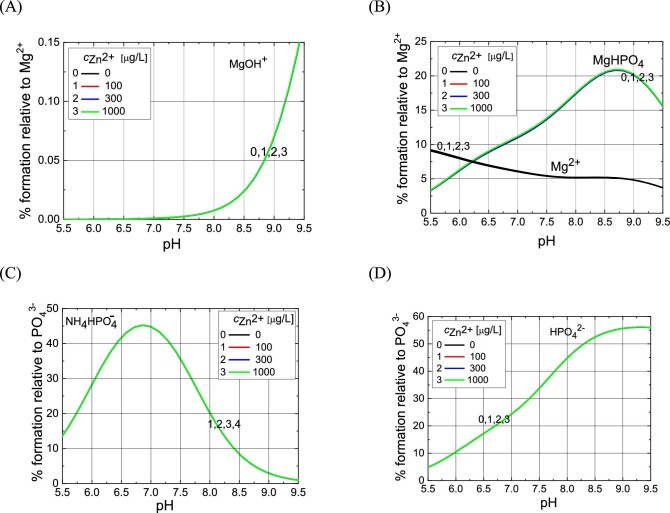
Percentage content of various Mg^2+^ complexes (A and B), free Mg^2+^ ions (B) and PO_4_^3−^ complexes (C and D) depending on pH of artificial urine for different Zn^2+^ concentrations given in the insets. The percentage content is given with respect to the initial concentration of Mg^2+^ ion (A and B) and PO_4_^3^^−^ (C and D) given in Table 5. All curves overlap at different Zn^2+^ concentrations, which means that the Zn^2+^ ion at concentrations used in the experiment has practically no effect on the percentage content of these chemical complexes. It should be noted that the concentrations of Zn^2+^ ions are very low compared to other components of artificial urine (see Table 5).

**Fig. 9:**
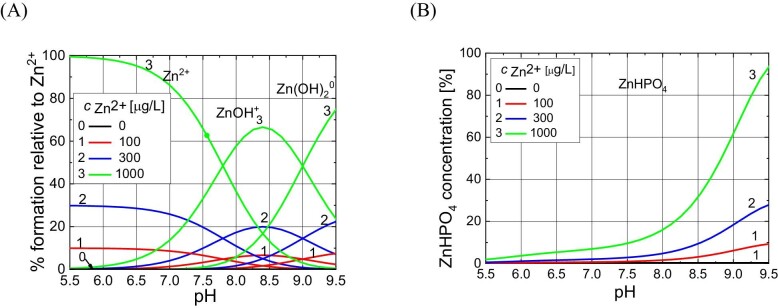
Percentage content of various Zn^2+^ hydroxo-complexes (A) and ZnHPO_4_ complex (B) depending on pH of artificial urine for different Zn^2+^ concentrations given in the insets.

**Fig. 10:**
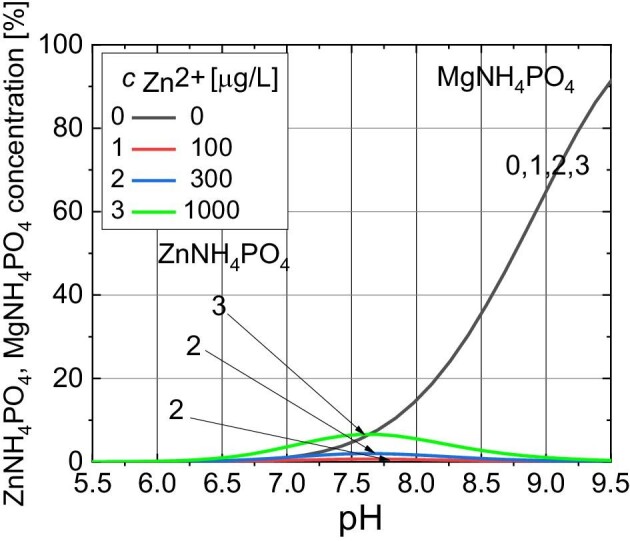
Percentage content of complexes MgNH_4_PO_4_ and ZnNH_4_PO_4_ versus pH of artificial urine for different Zn^2+^ concentrations given in the inset. The curves for the MgNH_4_PO_4_ complex overlap at different Zn^2+^ concentrations, which means that the Zn^2+^ ion at concentrations used has no effect on the percentage content of this complex. It should be noted that the concentration of Zn^2+^ ions is very low compared with other artificial urine components (see Table 5).

After entering the initial ion concentrations (Table 5) and the stability constants and solubility product constants of the formed complexes (Table 6) into the HySS computer program, the appropriate molar and percentage concentrations of chemical complexes in the given pH range were calculated. The results of this analysis, i.e. concentrations of dominant complexes in artificial urine for the baseline and for samples with the addition of Zn^2+^ ions, are presented in Figs. 8–10.

**Table 6.** Stability constants—log*β* of chemical complexes which are considered in the analysis. The presented stability constants are calculated using computer code EQUIL^44^ at 37 °C

Complex	−log*β*	Complex	−log*β*
HPO_4_^2−^	12.6	MgC_2_O_4_	3.62
H_2_PO_4_^−^	19.3	Mg_2_C_2_O_4_^2+^	4.28
H_3_PO_4_	21.56	Mg(C_2_O_4_)_2_^2−^	4.38
NaHPO_4_^−^	13.2	NH_4_C_2_O_4_^−^	1.11
KHPO_4_^−^	13.3	H_2_C_2_O_4_	5.68
MgCit^−^	4.67	MgOH	3.16
MgHCit	13.07	Mg_4_OH_4_^4+^	16.43
MgH_2_Cit^+^	12.43	KOH	1.5
MgHPO_4_	15.16	HSO_4_^−^	2.14
MgPO_4_^−^	5.88	NaSO_4_^−^	0.61
MgH_2_PO_4_^+^	20.53	KSO_4_^−^	0.997
NH_4_HPO_4_^−^	13.16	NH_4_SO_4_^−^	0.67
MgNH_4_PO_4_	0.0043	NH_4_OH	6.732
KMgPO_4_	1	MgSO_4_	2.407
Mg_3_(PO_4_)_2_	1	KC_2_O_4_^−^	1.2
HCit^2−^	6.46	KCit^2−^	1.24
H_2_Cit^−^	11.16	NH_4_Cit^2−^	0.085
H_3_Cit	14.49	HC_2_O_4_^−^	4.32
NaCit^2−^	1.35	ZnNH_4_PO_4_	0.0043
Zn(OH)^+^	6.2^b^	NaC_2_O_4_^−^	0.995
Zn(OH)_2_	11.2^b^	Zn(OH)_4_^2−^	15.57^b^
Zn(OH)_3_^−^	13.9^b^	ZnCit^−^	11.4^a^
ZnHPO_4_	14.4^a^	ZnHCit	20.8^a^
ZnSO_4_	2.3^a^	ZnH_2_Cit^+^	25^a^
ZnC_2_O_4_	3.9^a^	ZnHC_2_O_4_^2+^	5.5^a^
Zn(C_2_O_4_)_2_^2−^	6.4^a^	ZnH_2_(C_2_O_4_)_2_	10.7^a^

a Ref.^55^

b Ref.^56^

First, we perform speciation analysis to explain why in the presence of Zn^2+^ ions, struvite is formed earlier, i.e. at lower pH, compared with baseline (Fig. 2). It should be noted that the concentration of Mg^2+^ ions in urine (resulting from the composition of urine, Table 1) is 3.2 mM. The concentration of Zn^2+^ ions compared with the concentration of Mg^2+^ ions is low and amounts to 1.53×10^−3^, 4.58×10^−3^, and 15.3×10^−3^ mM (Table 5). This means that the ratio of Zn^2+^ to Mg^2+^ ions in the tested samples is: 1:2.091; 1:698; and 1:209.

In the pH range where Mg-struvite is formed, Mg^2+^ ions occur in free form or in the form of various complexes, e.g. MgOH^+^, MgHPO_4_ (Fig. 8A and B). Figure 8A and B shows that all curves overlap regardless of the Zn^2+^ concentration. This means that the change in the percentage content of these complexes is unnoticeable despite the fact that Zn^2+^ ions enter into various chemical reactions. However, it should be noted that the concentration of Zn^2+^ ions is very low compared with other components of artificial urine, as highlighted in the paragraph above. The situation is similar with the NH_4_HPO_4_^−^ and HPO_4_^2−^ complexes (Fig. 8C and D), i.e. their percentage content in the presence of Zn^2+^ does not differ from that for the control test (concentration 0).

As illustrated in Fig. 8A, at pH around 7.2, the amount of the MgOH^+^ complex increases significantly, regardless of the concentration of Zn^2+^ ions. At the same time, Fig. 8C shows that the highest concentration of the NH_4_HPO_4_^−^ complex occurs around pH 7, also regardless of the concentration of Zn^2+^ ions. This means that one of the possible reactions leading to the formation of struvite, in all cases examined, i.e. without and in the presence of Zn^2+^ ions, is the following reaction:


(1) \begin{eqnarray*}{\mathrm{N}}{{{\mathrm{H}}}_4}{\mathrm{HP}}{{{\mathrm{O}}}_4}^ - + {\mathrm{MgO}}{{{\mathrm{H}}}^ + } \to {\mathrm{MgN}}{{{\mathrm{H}}}_4}{\mathrm{P}}{{{\mathrm{O}}}_4} + {{{\mathrm{H}}}_2}{\mathrm{O}}\end{eqnarray*}

Figure 9 illustrates the percentage content of hydroxo-complexes formed by Zn^2+^, in the pH range from 5.5 to 9.5. These are the following hydroxo-complexes: ZnOH^+^, Zn(OH)_2_^0^, and Zn(OH)_3_^−^. Figure 9 shows that in this case, the percentage content of complexes formed depends on the concentration of Zn^2+^ ions and their maximum percentage content occurs at pH around 8.3, regardless of the concentration of Zn^2+^ ions. Zinc ions in the artificial urine enter into a hydrolysis reaction, releasing H^+^ ions:


(2) \begin{eqnarray*}{\mathrm{Z}}{{{\mathrm{n}}}^{2 + }} + {{{\mathrm{H}}}_2}{\mathrm{O}} \leftrightarrow {\mathrm{ZnO}}{{{\mathrm{H}}}^ + } + {{{\mathrm{H}}}^ + }\end{eqnarray*}

H^+^ ions formed as a result of the hydrolysis reaction are neutralized, among others, by adding an aqueous ammonia solution (NH_3_·H_2_O):


(3) \begin{eqnarray*}{\mathrm{N}}{{{\mathrm{H}}}_3} \cdot {{{\mathrm{H}}}_2}{\mathrm{O}} + {{{\mathrm{H}}}^ + } \leftrightarrow {\mathrm{N}}{{{\mathrm{H}}}_4}^ + + {{{\mathrm{H}}}_2}{\mathrm{O}}\end{eqnarray*}

As a reminder, an aqueous ammonia solution NH_3_·H_2_O is added to artificial urine to simulate the presence of bacteria and the decomposition of urea as described in section ‘Study of struvite crystallization in artificial urine without and with bacteria’.

As Fig. 9A shows, in the pH range in which struvite is formed, in the presence of zinc ions, mainly ZnOH^+^ and Zn(OH)_2_^0^ hydroxo-complexes are formed. ZnHPO_4_ is also formed (Fig. 9B). In other words, Zn^2+^ ions compete with Mg^2+^ ions. However, as shown in Fig. 8, this competition does not significantly affect the concentrations of complexes with magnesium ions because the concentrations of Zn^2+^ ions are very low compared with the concentration of Mg^2+^ ions. However, in the presence of Zn^2+^ ions, a competitive reaction to reaction (1) shown below may occur:


(4) \begin{eqnarray*}{\mathrm{N}}{{{\mathrm{H}}}_4}{\mathrm{HP}}{{{\mathrm{O}}}_4}^ - + {\mathrm{ZnO}}{{{\mathrm{H}}}^ + } \to {\mathrm{ZnN}}{{{\mathrm{H}}}_4}{\mathrm{P}}{{{\mathrm{O}}}_4} + {{{\mathrm{H}}}_2}{\mathrm{O}}\end{eqnarray*}

This means that co-precipitation of Mg-struvite (MgNH_4_PO_4_ complex) and Zn-struvite (ZnNH_4_PO_4_ complex) can occur. Figure 10 illustrates the formation of these complexes. Such co-precipitation consists in replacing Mg^2+^ ions in the struvite crystal lattice with Zn^2+^ ions. Reaction (4), i.e. the formation of the ZnNH_4_PO_4_ complex, which is synonymous with the formation of Zn-struvite, can occur at slightly lower pH values than reaction (1), i.e. Mg-struvite precipitation, because ZnOH^+^ complexes (Fig. 9A) are formed at lower pH than MgOH^+^ complexes (Fig. 8A).

As mentioned above, in the pH range at which Mg-struvite is formed, there are various ions and complexes i.e. Mg^2+^, Zn^2+^, MgHPO_4_, ZnHPO_4_, HPO_4_^2−^ (see Figs. 8–10). Thus, other Mg-struvite formation reactions can be written as follows:


(5) \begin{eqnarray*}{\mathrm{M}}{{{\mathrm{g}}}^{2 + }} + {\mathrm{N}}{{{\mathrm{H}}}_3} \cdot {{{\mathrm{H}}}_2}{\mathrm{O}} + {\mathrm{HP}}{{{\mathrm{O}}}_4}^{2 - } \to {\mathrm{MgN}}{{{\mathrm{H}}}_4}{\mathrm{P}}{{{\mathrm{O}}}_4} + {{{\mathrm{H}}}_2}{\mathrm{O}}\end{eqnarray*}

(6) \begin{eqnarray*}{\mathrm{MgHP}}{{{\mathrm{O}}}_4} + {\mathrm{N}}{{{\mathrm{H}}}_3} \cdot {{{\mathrm{H}}}_2}{\mathrm{O}} \to {\mathrm{MgN}}{{{\mathrm{H}}}_4}{\mathrm{P}}{{{\mathrm{O}}}_4} + {{{\mathrm{H}}}_2}{\mathrm{O}}\end{eqnarray*}

and competitive to reactions (5) and (6), presented below:


(7) \begin{eqnarray*}{\mathrm{Z}}{{{\mathrm{n}}}^{2 + }} + {\mathrm{N}}{{{\mathrm{H}}}_3}\! \cdot \! {{{\mathrm{H}}}_2}{\mathrm{O}} + {\mathrm{HP}}{{{\mathrm{O}}}_4}^{2 - } \to {\mathrm{ZnN}}{{{\mathrm{H}}}_4}{\mathrm{P}}{{{\mathrm{O}}}_4} + {{{\mathrm{H}}}_2}{\mathrm{O}}\end{eqnarray*}

(8) \begin{eqnarray*}{\mathrm{ZnHP}}{{{\mathrm{O}}}_4} + {\mathrm{N}}{{{\mathrm{H}}}_3}\! \cdot \! {{{\mathrm{H}}}_2}{\mathrm{O}} \to {\mathrm{ZnN}}{{{\mathrm{H}}}_4}{\mathrm{P}}{{{\mathrm{O}}}_4} + {{{\mathrm{H}}}_2}{\mathrm{O}}\end{eqnarray*}

To sum up, the results of the speciation analysis of the formation of chemical complexes indicate that in the presence of Zn^2+^ ions in the artificial urine, MgNH_4_PO_4_ and ZnNH_4_PO_4_ complexes are formed, which enable the formation of Mg-struvite and Zn-struvite, respectively. As can be seen from Fig. 10, ZnNH_4_PO_4_ complexes are formed at lower pH values compared with MgNH_4_PO_4_ complexes. Additionally, the higher the concentration of Zn^2+^ ions, the greater the shift in the formation of ZnNH_4_PO_4_ complexes towards lower pH. This result suggests a conclusion regarding the results presented in Fig. 2, which show that the higher the concentration of Zn^2+^, the earlier, i.e. for the lower pH, crystalline phases are formed. Based on the speciation analysis, the obtained experimental results (Fig. 2) can be explained as follows: in the presence of Zn^2+^ ions, Mg^2+^ ions are successively replaced with Zn^2+^ ions in the crystal lattice, which means the formation of Zn*_x_*-struvite. As shown by the analyses of chemical complexes, ZnNH_4_PO_4_ complexes involved in the formation of Zn*_x_*-struvite are formed earlier, i.e. at a lower pH, which is consistent with the experimental results (Fig. 2). These results are also confirmed by XRD tests, Fig. 7.

### Effect of Zn^2+^on nucleation and growth of struvite in the presence of urease-positive bacteria

#### Spectrophotometric analysis

**Fig. 11:**
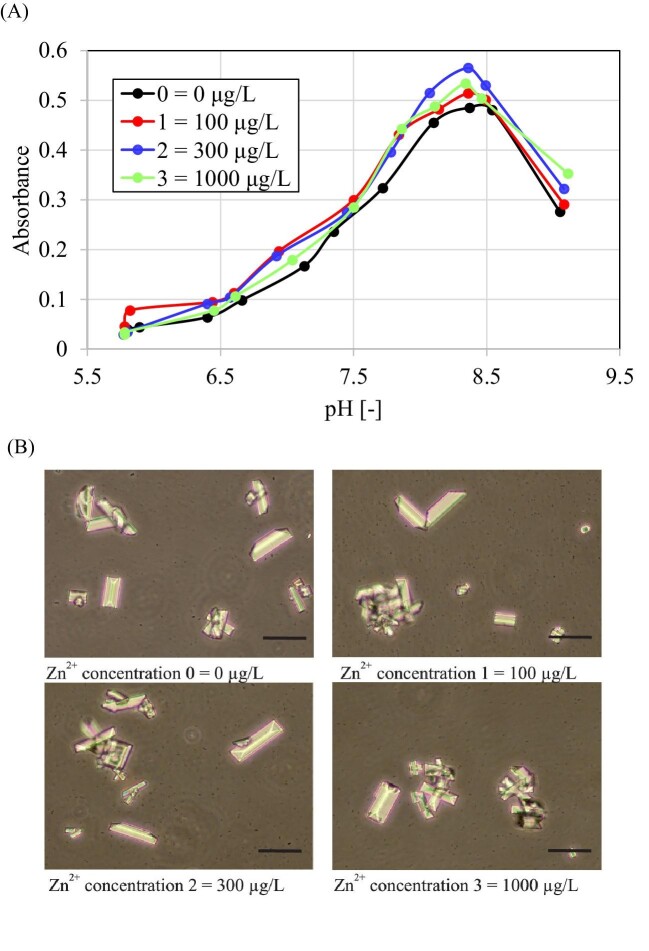
(A) Absorbance of artificial urine versus pH for various Zn^2+^ concentrations (top row) in the presence of bacteria. (B) habits of struvite crystals 24 h after adding bacteria to the samples; scale bar 50 μm.

In the presence of bacteria, the progress of struvite nucleation and growth was monitored for the first 8 h after incubation of bacteria in artificial urine, and then after 24 h after this incubation. The results of spectrophotometric measurements are shown in Fig. 11A.

Based on Fig. 11A, it can be concluded that the absorbance started to increase slightly earlier (at lower pH) for any of the zinc ion-containing samples (samples 1, 2, and 3) compared with the baseline (sample 0, no Zn^2+^ ion) as this was observed in an experiment without the presence of bacteria (Fig. 2). This is related to the simultaneous crystallization of Mg-struvite and Zn*_x_*-struvite. As speciation analysis indicates, the Zn^2+^ hydroxo-complexes that then build Zn-struvite, e.g. according to reactions (4), (7), or (8), are formed earlier, i.e. at a lower pH. Hence, the slight shift in the formation of crystalline precipitate towards lower pH for samples containing Zn is attributed, as in the case of the experiment without bacteria, to the formation of Zn-struvite. In addition, the maximum absorbance values at pH = 8.4 are higher for zinc-containing samples compared with the baseline test. This may mean that more struvite crystals are formed for zinc-containing samples in the presence of bacteria. It should be emphasized that a significant decrease in absorbance for the highest pH, observed after 24 h of the experiment, is related to the sedimentation of struvite crystals. The sedimentation rate is directly proportional to the size and mass of the particles dispersed in the solution, i.e. it is faster for larger and heavier particles. After 24 h of the experiment, the struvite crystals are larger and heavier and sediment quickly, certainly faster than the duration of the absorbance measurement.

Figure 11B shows the habit of struvite crystals. It can be seen that in the baseline test, the struvite assumes a characteristic coffin-like habit, similar to that which occurs in the absence of bacteria. This habit has been repeatedly described in the literature.^46–51^ When a Zn^2+^ ion is present (Fig. 11B), struvite does not change habit and morphology, even at higher concentrations of this ion. As in the case of the experiment without bacteria, also in this case, penetration twins with a shape similar to X are observed. These twins are formed by crystals of smaller sizes compared with those that do not form twins.

**Fig. 12:**
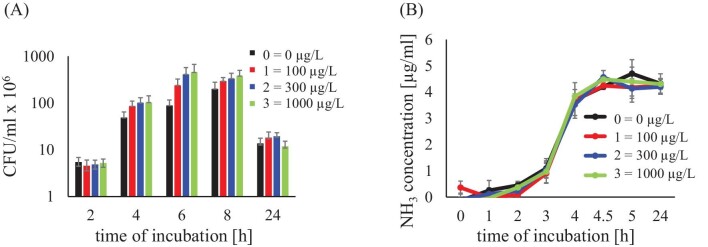
The effect of Zn^2+^ ions on the number of bacteria (A) and the activity of their enzyme—urease (B) in various concentrations of Zn^2+^ ions.

Zn^2+^ may also affect bacterial viability. Therefore, the number of bacteria was determined in each sample after different incubation times. As shown in Fig. 12A, the number of bacteria increases in all samples up to 6 h, but at 24 h, it is much lower, which is due to the unfavourable effect of high pH on bacteria. As mentioned earlier, the pH level is correlated with the viability of the bacteria. Except for the test after 24 h, in the case of samples containing Zn^2+^, the number of bacteria is higher than in the control test, and bacterial growth intensifies with the increase in the concentration of this ion. The question arises whether this is related to bacterial activity. The measure of the activity of bacteria, or rather bacterial urease, is the amount of ammonia, due to the fact that it is formed after the decomposition of urea by bacterial urease.^9–14^ Therefore, Fig. 12B shows the amount of ammonia released as a result of urease activity. The course of the curves indicates that in each case the ammonium content is comparable regardless of the presence of zinc. This means that the increase in the number of bacteria is not due to the increased urease activity of these bacteria. Hence, the conclusion that the observed increase in the number of bacteria should be attributed to zinc, which must have a beneficial effect on bacteria and their viability.

A literature review shows that zinc may be toxic and has a bactericidal effect on bacteria, which is why it has been proposed for the treatment of various bacterial infections, including urinary tract infections.^57,58^ Zinc is also necessary for the life of bacteria. It is associated with ∼5% of bacterial proteins and many important enzymes require the zinc ion for their activity. Zinc is involved in the control of bacterial metabolism and cellular transport.^59^ The effect of zinc on bacteria depends on its concentration and the individual sensitivity of the bacteria to this ion. In the case of the presented studies, zinc in the concentrations used is not toxic to *P. mirabilis* and, by positively influencing cellular metabolism, enables better bacterial growth.

In order to check the intensity of crystallization and the zinc content in the resulting crystals, in the case of experiments with bacteria, the presence of magnesium and zinc in the overall sediment containing both bacterial suspension and crystals was assessed (see section ‘Study of struvite crystallization in artificial urine without and with bacteria’). Samples were analysed after 5 and 24 h of incubation. The amount of magnesium after 24 h (Table 7), which indicates the amount of struvite formed, is the highest for zinc concentration 1, slightly lower for concentration 2 and the lowest for concentration 3. This result correlates well with the mass of crystalline precipitate formed without the presence of bacteria—Table 2. Regarding Zn^2+^, it is worth noting that for the control test (without Zn^2+^), Zn^2+^ is present in the tested samples (Table 7). This may be surprising because artificial urine (Table 1) does not contain any zinc-containing chemical compound. Therefore, the presence of zinc in the control samples (without the addition of Zn^2+^ ions) should be attributed to TSB (Table 1), a component added only in the presence of bacteria. TSB is a nutritious medium used to grow microorganisms, including bacteria. This substrate provides amino acids, nitrogen, carbon, vitamins, and minerals necessary for the growth of organisms. Additionally, TSB usually contains sodium chloride, which maintains osmotic balance in the medium, natural sugars, which are an energy source, and dipotassium phosphate, which is a buffering agent.^60^ Despite our efforts, we have not found any literature sources that would explicitly state that TSB contains zinc. However, in our samples, the only possible source of zinc in control samples is TSB. On the other hand, it should be noted that the Zn^2+^ concentration in the control samples is 0.28 and 0.22 μg/ml after 5 and 24 h of experiment, respectively. Compared to the concentration of, for example, magnesium, the concentration of zinc in control samples is 170 times lower. However, it should be noted that the level of Zn^2+^ in samples with concentrations 1, 2 and 3 after 5 h of the experiment is higher compared to the control sample, and after 24 hours the level of Zn^2+^ is higher for samples with concentrations 2 and 3. Due to the fact that the zinc content was determined in this case in the final sediment containing both the bacterial suspension and crystals, the presence of zinc in these samples may be related to both the presence of zinc in the crystals and the accumulation of this ion in bacterial cells. The accumulation of Zn^2+^ ions in bacterial cells can be evidenced by the higher zinc content in samples after 5 h (Table 7), for which the number of bacteria is higher (Fig. 12A) compared to samples after 24 h. Some of the remaining Zn^2+^ ions, to some extent, replaced the magnesium ions in the struvite crystal lattice to form Zn*_x_*-struvite, similar to the experiment without bacteria.

**Table 7.** Zn^2+^ and Mg^2+^ ions concentrations in sediments (bacteria and crystals) obtained from samples in artificial urine containing different concentrations of zinc ion

	Concentration [µg/ml ± SD*]			
	Zn^2+^		Mg^2+^	
Sample	5 h	24 h	5 h	24 h
0 = 0 µg/l, without Zn^2+^; baseline	0.28 ± 0.05	0.22 ± 0.04	47.5 ± 3.5	55.6 ± 4.6
with Zn^2+^				
1 = 100 µg/l	0.34 ± 0.05	0.22 ± 0.08	44.5 ± 3.8	57.3 ± 4.1
2 = 300 µg/l	0.31 ± 0.06	0.26 ± 0.07	43.8 ± 4.1	54.3 ± 4.3
3 = 1000 µg/l	0.33 ± 0.08	0.28 ± 0.07	40.1 ± 3.3	51.0 ± 3.1

*SD means standard deviation.

## Conclusions

The conducted research shows that in artificial urine, Zn^2+^ ions compete with Mg^2+^ ions. This competition leads to the replacement, to some extent, of Mg^2+^ ions in the struvite crystal lattice with Zn^2+^ ions. This means that there is co-precipitation of Mg-struvite (MgNH_4_PO_4_·6H_2_O) and Zn*_x_*-struvite (Mg_1-_*_x_*Zn*_x_*NH_4_PO_4_·6H_2_O). Zn*_x_*-struvite can precipitate at slightly lower pH values than Mg-struvite because, as indicated by speciation analysis, ZnOH^+^ complexes are formed at lower pH than MgOH^+^ complexes. The results of the speciation analysis validate the XRD results of the tested samples, which indicate that Mg^2+^ ions are indeed exchanged, to some extent, for Zn^2+^ ions. These results are consistent with the experimental results, which indicate that in the presence of Zn^2+^ ions, crystalline solid phases are formed earlier (at lower pH) compared to the control test (without Zn^2+^ ions). In other words, the Zn^2+^ ions shift the nucleation point of the crystalline solids towards a lower pH. In the presence of bacteria, the results are similar, i.e. in the presence of Zn^2+^ ions, earlier (for lower pH) precipitation of crystalline solid phases is observed compared to the control test (without Zn^2+^ ions). Additionally, in the case of samples containing zinc, the number of bacteria is higher than in the control test, and this number increases with the increase in the concentration of this ion. The conducted research does not indicate that this effect is related to the activity of bacterial urease, therefore we attribute this effect to zinc, which must have a beneficial effect on bacteria and their viability. In a broader perspective, the obtained results indicate that zinc ions Zn^2+^ in the range of tested concentrations, in the case of urinary tract infection with urease-positive bacteria, may slightly increase the risk of developing infection urinary stones.

Zinc ions also influence the absolute size of struvite crystals. For the control sample (without Zn^2+^) the crystals have the largest average absolute dimensions compared to the zinc-containing samples. With the increasing concentration of Zn^2+^ ions in the tested samples (Zn^2+^ concentration 1 and 2), a clear decrease in the absolute dimensions of the crystals can be observed. Only for the extreme concentration of Zn^2+^ (1000 μg/l) there is a slight change in this trend. In addition to the absolute dimensions, with increasing Zn^2+^ concentration, the aspect ratio, *AR*, of the crystals, also changes. This means that Zn^2+^ ions also influence the relative growth rates of individual struvite crystal faces.

As mentioned in the Introduction, meat contains large amounts of Zn, and additional literature sources ^25,61,62^ indicate that Zn absorption is much greater in the presence of protein contained in meat. Therefore, it can be hypothesized that this may contribute to the increase in the incidence of infection urinary stones in highly developed countries, because meat consumption in these countries is very high. For example, in Australia or the USA, meat consumption reaches 100 kg/capita/year, while in India or Ethiopia, meat consumption is 5 kg/capita/year.^63^

## Data Availability

All data are incorporated into the article.
